# Inhibition of Homophilic Interactions and Ligand Binding of the Receptor for Advanced Glycation End Products by Heparin and Heparin-Related Carbohydrate Structures

**DOI:** 10.3390/medicines5030079

**Published:** 2018-07-30

**Authors:** Ari Rouhiainen, Niko-Petteri Nykänen, Juha Kuja-Panula, Päivi Vanttola, Henri J. Huttunen, Heikki Rauvala

**Affiliations:** 1 Neuroscience Center, University of Helsinki, 00014 Helsinki, Finland; Niko-Petteri.Nykaenen@dzne.de (N.-P.N.); juha.kuja-panula@helsinki.fi (J.K.-P.); paivant@gmail.com (P.V.); henri.huttunen@helsinki.fi (H.J.H.); heikki.rauvala@helsinki.fi (H.R.); 2 Department of Biosciences, University of Helsinki, 00014 Helsinki, Finland; 3 Department of Translational Neurodegeneration, German Center for Neurodegenerative Diseases (DZNE), D-81377 Munich, Germany; 4 Faculty of Medicine, University of Helsinki, 00014 Helsinki, Finland

**Keywords:** dimerization, glycyrrhizin, heparin, HMGB1, K5, RAGE

## Abstract

**Background:** Heparin and heparin-related sulphated carbohydrates inhibit ligand binding of the receptor for advanced glycation end products (RAGE). Here, we have studied the ability of heparin to inhibit homophilic interactions of RAGE in living cells and studied how heparin related structures interfere with RAGE–ligand interactions. **Methods:** Homophilic interactions of RAGE were studied with bead aggregation and living cell protein-fragment complementation assays. Ligand binding was analyzed with microwell binding and chromatographic assays. Cell surface advanced glycation end product binding to RAGE was studied using PC3 cell adhesion assay. **Results:** Homophilic binding of RAGE was mediated by V_1_- and modulated by C_2_-domain in bead aggregation assay. Dimerisation of RAGE on the living cell surface was inhibited by heparin. Sulphated K5 carbohydrate fragments inhibited RAGE binding to amyloid β-peptide and HMGB1. The inhibition was dependent on the level of sulfation and the length of the carbohydrate backbone. α-d-Glucopyranosiduronic acid (glycyrrhizin) inhibited RAGE binding to advanced glycation end products in PC3 cell adhesion and protein binding assays. Further, glycyrrhizin inhibited HMGB1 and HMGB1 A-box binding to heparin. **Conclusions:** Our results show that K5 polysaccharides and glycyrrhizin are promising candidates for RAGE targeting drug development.

## 1. Introduction

The receptor for advanced glycation end products (RAGE) is a multiligand cell surface transmembrane receptor that regulates normal lung physiology, cell motility, and inflammatory reactions. RAGE and some of its ligands (HMGB1, S100A8/S100A9, and amyloid β-peptide) bind to proteoglycans on the cell surface and these interactions regulate the ligand binding and signaling of the receptor [1,2].

RAGE homodimerisation and oligomerisation occurs in cells [3]. Homophilic interactions of RAGE in living cells is demonstrated with fluorescence resonance energy transfer and bioluminescence resonance energy transfer methods [4,5]. Formation of RAGE homodimers and oligomers in solution is shown using various biochemical assays including cross-linking, nuclear magnetic resonance, and X-ray crystallography methods [6,7].

Homodimerization and oligomerisation of RAGE can be regulated by receptor ligand binding. In solution, heparin enhances dimer formation of soluble RAGE. Cleavage of heparan sulphate chains on the cell surface inhibits RAGE signaling, suggesting that RAGE forms complexes with proteoglycans [2]. In addition, homophilic interactions of RAGE can be regulated by S100 protein and advanced glycation end product bovine serum albumin (AGE-BSA) ligands. Ligand induced receptor dimerisation and oligomerisation is required for strong RAGE signaling [6,7].

α-d-Glucopyranosiduronic (glycyrrhizin) is a triterpenoid saponin derived from liquorice root. It is a well-tolerated drug in humans and it has well-documented hepatoprotective effects in the treatment of chronic hepatitis B and C infections [8,9,10,11]. Intravenous glycyrrhizin downregulates serum aspartate aminotransferase and alanine transaminase levels and reduces the occurrence of hepatocellular carcinoma. Further, glycyrrhizin has numerous other protective pharmacological functions, like anti-ulcer and anti-allergic effects (reviewed in the literature [12,13]). Glycyrrhizin has anti-viral effects in vitro and it dampens inflammatory reactions in various animal models [14,15,16,17,18,19]. Anti-inflammatory effects of glycyrrhizin are at least partly due to its ability to downregulate proinflammatory cytokine expression and inhibit leukocyte diapedesis. Glycyrrhizin and heparin have d-glucuronic acid units in their structure and they both are potent inhibitors of transendothelial migration of leukocytes [20]. However, in heparin, d-glucuronic acid units are mostly C5-epimerised to L-iduronic acid and are highly sulphated. Glycyrrhizin can be chemically sulphated, which potentiates its anti-viral effects [21,22].

Both glycyrrhizin and heparin bind to HMGB1 and inhibit cell adhesion and migration to HMGB1 [23,24,25,26,27,28]. HMGB1 mediates cell migration via both RAGE independent and dependent mechanisms [29]. RAGE independent migration of cells can occur via HMGB1—stromal cell-derived factor 1 (SDF-1) heterocomplex mediated pathway [28]. SDF-1 signals through C–X–C chemokine type receptor 4 (CXCR4). In addition, SDF-1 binding to heparan sulphate proteoglycan (HSPG) type cell surface receptors is a prerequisite to it promoting activity of tissue emigration of cells [30]. Further, the importance of HMGB1 in cell migration in vivo was shown in the study by Oyama and others, where decreased leukocyte diapedesis occurred in HMGB1 heterozygote mice [31]. It has been unclear whether interactions of HMGB1 with HSPGs, which mediate cell adhesion and migration, can be inhibited by glycyrrhizin.

Here, we have studied the targeting of RAGE and RAGE ligands by heparin and heparin-related structures.

## 2. Materials and Methods

### 2.1. Materials

Recombinant HMGB1 was produced as described [32]. The extracellular coding regions of human RAGE were cloned into the modified pRMHA3 vector containing the CD33 signal sequence and human IgG Fc-part. Immunoglobulin fusion proteins were produced in Drosophila S2 cells and purified using protein A-agarose chromatography as described [33,34]. Full length extracellular human RAGE (sRAGE) contained amino acids 28–331; V_1_-domain amino acids 28–119; C_1_-domain amino acids 120–231; C_2_-domain amino acids 232–331; delta V_1_ amino acids 120–331; delta C_1_ amino acids 28–119 + 232–331; delta C_2_ amino acids 28–231. AGE-BSA was produced as described [35]. Recombinant HMGB1 A-box (amino acids 1–84) was produced as a glutathione S-transferase (GST) fusion protein and purified using the same method as described for deltaC-HMGB1 purification [35]. Amyloid β peptide 1–42 was from American Peptide (Sunnyvale, CA, USA). K5 carbohydrates, chondroitin sulphate, and dermatan sulphate were from Iduron (Manchester, United Kingdom). Composition of K5 carbohydrates is described in the literature [36]. Glycyrrhizin (Sigma-Aldrich, St. Louis, MO, USA) and glucose stock solutions (0.1–0.25 M) were made in 1% ethanol, pH 7.5 [37]. Salt concentrations in glycyrrhizin and glucose stocks and in buffer controls were equalized. For PC3 adhesion studies, glycyrrhizin or glucose were dissolved in a NaOH solution as described [38]. Heparin was from Sigma-Aldrich.

### 2.2. Bead Aggregation Assay

Protein A coated bead aggregation mediated by recombinant Ig-fusion proteins was studied as described [33]. In brief, monomeric fluorescent protein A coated beads were incubated with 10 μg/mL of different sRAGE–Ig forms and aggregation was monitored using fluorescence microscopy (Olympus, Tokyo, Japan). Pictures were taken after 60 min bead aggregation. The extent of bead aggregation is represented by the index N_t_/N_0_. N_t_ and N_0_ are the total number of particles at incubation times t and 0, respectively.

### 2.3. Live-Cell Protein-Fragment Complementation Assay (PCA)

Full-length RAGE was cloned to plasmids containing complementary fragments of humanized Gaussia (hGLuc) luciferase and livecell protein-fragment complementation assay was done as described [39]. In brief, white-walled 96-well plates (Perkin Elmer, Waltham, MA, USA) were coated with poly-l-lysine (Sigma), and 10,000 cells per well were seeded. Transfection of the phGLuc–RAGE plasmids (100 ng of DNA per well) was performed after 24 h, while detection of PCA signal was measured 48 h post-transfection. Culture media was changed 30 min before measurements to serum and phenol red-free Dulbecco’s Modified Eagle Medium (DMEM) (Invitrogen, Carlsbad, CA, USA). Luminescence was measured with Victor^3^ plate reader (Perkin Elmer) following injection of native coelenterazine, the substrate for the GLuc (NanoLight Technology, Pinetop, AZ, USA). At least four replicate wells per condition were analyzed and a minimum of three independent experiments were performed.

### 2.4. Binding of Amyloid Beta 1–42 Peptide to RAGE

Ig-fusion protein of sRAGE in a concentration of 5 mg/mL was bound to protein A (1 mg/mL, Sigma-Aldrich, St. Louis, MO, USA) coated wells (Maxisorb ELISA plates, Sigma-Aldrich). Unbound proteins were washed away, and the wells were blocked with BSA (Sigma-Aldrich). Biotinylated amyloid β 1–42 peptide (20 nM, American Peptide, Sunnyvale, CA, USA) or biotinylated HMGB1 (20 nM) were bound to the wells in the presence or absence of inhibitors, and unbound ligands were washed away. Bound biotinylated ligands were detected with horse radish peroxidase conjugated streptavidin (Sigma-Aldrich) and peroxidase substrate (Sigma-Aldrich). The color was developed using *o*-phenylenediamine dihydrochloride peroxidase substrate (Sigma-Aldrich). Absorbance at 490 nm was measured.

### 2.5. RAGE Binding to AGE-BSA

Plastic Maxisorb microwells (Sigma-Aldrich) were coated with 20 μg/mL of AGE-BSA [35] and blocked with BSA. sRAGE–Ig protein (0.1 μg/mL) was incubated in wells in the presence of various amounts of glycyrrhizin. Bound proteins were quantified using horse radish peroxidase conjugated goat anti-human IgG (Sigma-Aldrich). The color was developed with peroxidase substrate. Absorbance was measured at 450 nm.

### 2.6. Cell Adhesion Studies

PC3 cell binding assay was carried out as described [40]. Briefly, Maxisorp (Sigma-Aldrich) wells were coated with RAGE V_1_–Fc, collagen, laminin, or fibronectin [10 μg/mL in phosphate buffered saline (PBS)]. Coated wells were washed with PBS, blocked with 0.5% BSA-PBS, and washed once with PBS. Ten microliters of Iscoves’s Modified Dulbecco’s Media (IMDM, Invitrogen)–0.5% BSA containing 11× concentration of possible inhibitors was added to wells, and 1 × 10^5^ PC3 cells in 100 μL of IMDM–0.5% BSA was added to wells. Cells were adhered for 30 min at 37 °C and unbound cells were washed away with warm IMDM–0.5% BSA (6 times) and bound cells were quantitated with intracellular acidic phosphatase assay [26]. EDTA, heparin, and glucose served as control inhibitors in cell adhesion studies.

### 2.7. HMGB1 Binding to Heparin

Heparin–Sepharose (GE Healthcare, Chicago, IL, USA) affinity chromatography of HMGB1 was carried out as previously described using PBS as a chromatography buffer [26]. HMGB1 was loaded to the column in the presence of different concentrations of glycyrrhizin, the column was washed with PBS, and bound protein was eluted with PBS containing 1.5 M NaCl. In some experiments, HMGB1 or thrombin were loaded onto the column in PBS and eluted with increasing amounts of glycyrrhizin in PBS. HMGB1 in elution fractions was detected with an anti-HMGB1 immunoblot [32], and thrombin was detected in a dot blot assay using Coomassie blue staining. The optical density (OD) values of bands or dots were measured as described [35].

The effect of glycyrrhizin on HMGB1 A-box binding to heparin–Sepharose was studied using heparin–Sepharose precipitation assay. The A-box was diluted with PBS (4 μg/mL) and an equal volume of 50% heparin–Sepharose slurry in PBS was added. Glycyrrhizin or glucose were at concentrations of 0–3 mM. Samples were incubated in rotation at 4 °C for 1 h, heparin–Sepharose was pelleted and washed with PBS. Bound protein was eluted with 30 μL of hot reducing SDS-PAGE sample buffer, and samples were analyzed using an anti-HMGB1 (anti peptide III, [32]) Western blotting assay. The OD values of A-box bands were measured.

Precipitation assays of full length HMGB1 protein. HMGB1 was diluted with PBS (25 μg/mL) and divided to 100 μL aliquots, and various amounts of glycyrrhizin or glucose were added. One-hundred microliters of 50% heparin–Sepharose slurry in PBS was added to aliquots, and the samples were incubated in rotation at 4 °C for 1 h. Heparin–Sepharose was washed once with PBS, and bound proteins were eluted with 30 μL of hot reducing SDS-PAGE sample buffer. The samples were run on SDS-PAGE and the gels were stained with Coomassie blue. The OD values of HMGB1 bands were measured.

### 2.8. Data Analyses

Analyses were performed using Microsoft Excel 2010 (Microsoft Corporation, Redmond, WA, USA), GraphPad Prism Software 4.0 (GraphPad Software Inc., La Jolla, CA, USA), and PSI-Plot V 7.0 (Poly Software International, Pearl River, NY, USA). Student’s *t*-test was used to compare group means. *p*-values < 0.05 were considered as significant.

## 3. Results and Discussion

### 3.1. Nature of Homophilic RAGE Interactions

Homophilic interactions of RAGE are mediated by multiple mechanisms involving both extra cellular and cytoplasmic domains [2,3,4,5,6,7]. Wei et al. have described non-covalent homophilic interactions occur between V_1_-domains and dimerization by covalent homophilic bonding via interchain disulphide bridge between C_2_-domains [3]. In addition, homophilic binding of soluble recombinant RAGE protein have been shown to require high concentrations of protein and the presence of divalent metal ions [41,42]. Here, we studied whether the homophilic interactions of recombinant sRAGE occur in low protein concentrations using bead aggregation assay. Furthermore, we studied whether the homophilic interactions of full length RAGE on a living cell surface can be inhibited by heparin.

**Figure 1:**
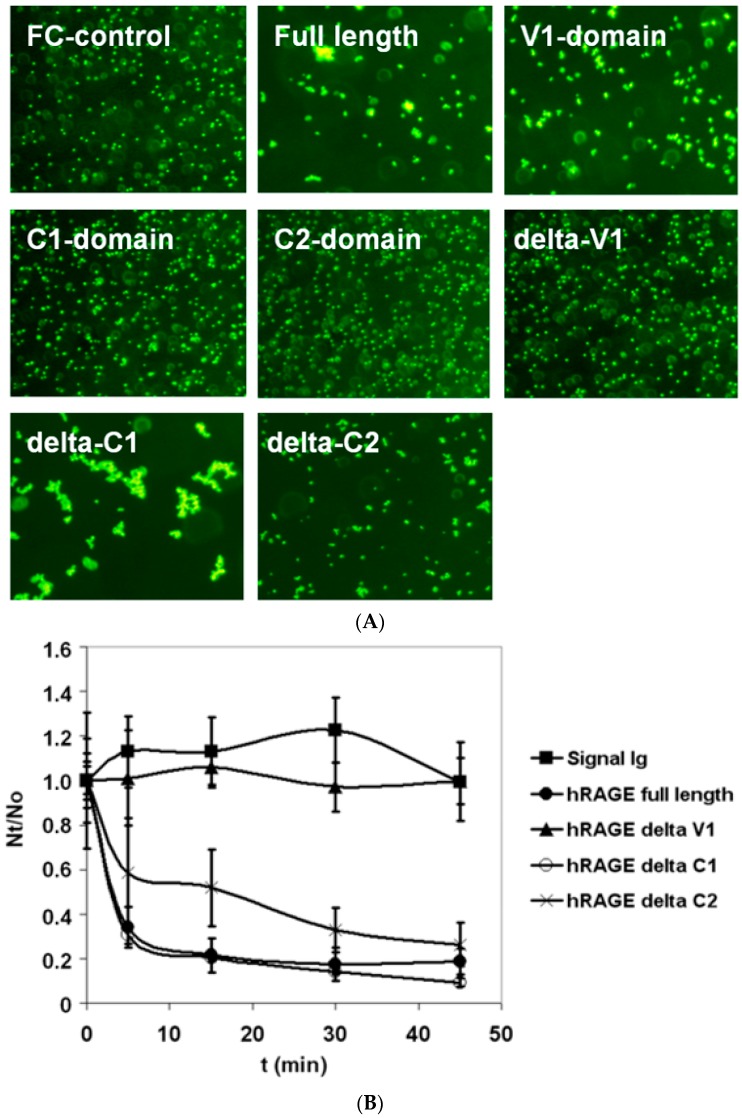
Bead aggregation assay of the receptor for advanced glycation end products (RAGE) homophilic binding. (**A**) Monomeric fluorescent protein A-coated beads were incubated with low concentration (10 μg/mL) of different Ig-tagged recombinant full length extracellular human RAGE (sRAGE) domains and aggregation was monitored using fluorescence microscopy. The images are taken after 60 min of bead aggregation. (**B**) Kinetics of bead aggregation. N_t_ and N_0_ are the total number of particles at incubation times t and 0, respectively. The extent of bead aggregation is represented by the index N_t_/N_0_. Closed circle curve represents full-length sRAGE–Ig fusion protein, close triangle curve represents sRAGE–Ig fusion protein lacking V_1_-domain, open circle curve represents sRAGE–Ig fusion protein lacking C_1_-domain, cross curve represents sRAGE–Ig fusion protein lacking C_2_-domain, and close square curve represents Fc control protein coated beads. Whereas V_1_-domain was necessary and sufficient for homophilic binding of RAGE, the existence of the C_2_-domain in recombinant sRAGE-fragment was required for maximal homophilic binding of RAGE forms bearing the V_1_- and C_2_-domains. N ≥ 3; error bars represent ±SD.

Recombinant sRAGE–Ig protein and Ig-fragments bearing RAGE domains used in this study are dimeric proteins. sRAGE–Ig and V_1_–Ig induced bead aggregation in protein-A coated bead suspension, suggesting that dimeric RAGE is able to form oligomeric structures in low concentrations (10 μg/mL) and in the absence of other exogenous ligands. The V_1_-domain was necessary and sufficient for observed oligomerisation. However, the lack of C_2_-domain in recombinant protein resulted in a diminished oligomerisation (Figure 1A,B).

**Figure 2:**
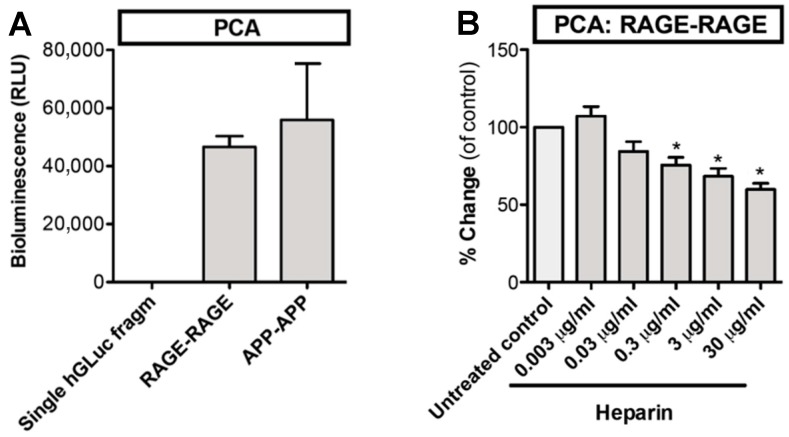
Homophilic interactions of RAGE on the living cell surface are inhibited by heparin. (**A**) Cells were transfected with full length RAGE–cytoplasmic hGLuc fragment chimeras. RAGE self-association was measured with luciferase activity. hDLuc fragment chimeras of APP served as a positive control of homodimerization. (**B**) RAGE self-association was partially inhibited by heparin. N = 3–4; error bars represent ±SD. * = *p* < 0.05 when compared with untreated control. hGLuc = humanized Gaussia luciferase, RLU = relative light unit, PCA = protein-fragment complementation assay, APP = amyloid β.

**Figure 3:**
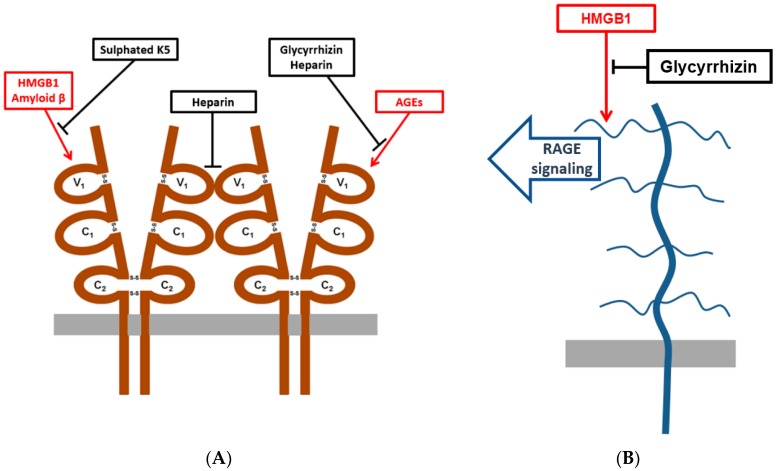
Schematic picture showing RAGE and HMGB1 antagonists used in this study. (**A**) A schematic picture showing RAGE extracellular V_1_-domain mediated interactions that were inhibited by glycyrrhizin, sulphated K5 oligosaccharides, and heparin in this study. (**B**) A schematic picture showing the predicted extracellular sulphated glycosaminoglycan binding of HMGB1 and inhibition of binding and RAGE signaling by glycyrrhizin.

Next, we studied the homophilic interactions of RAGE in living cells using a protein-fragment complementation assay (PCA). In this assay, cells are transfected with full-length RAGE–cytoplasmic Gaussia luciferase-fragment chimeras that become chemiluminescent when dimerised. Amyloid precursor protein (APP) that forms homodimers served as a positive control (Figure 2A). RAGE self-association occurred in living cells without any exogenous macromolecular ligands. Self-association was partially inhibited by heparin (Figure 2B). Our results indicate that the soluble dimeric ectodomain of RAGE, at low protein concentration and in the absence of exogenous ligands, can form oligomers. Further, to our knowledge, this is the first time that it has been shown that RAGE self-association in the living cells can be inhibited by heparin. As heparin does not penetrate through the plasma membrane, the inhibition most probably occurs at the cell surface. Xu et al. have shown that sRAGE dimerization is enhanced by heparin and dimerization at the cell surface is inhibited by heparanase treatment [2]. Our results suggest that soluble heparin may interfere with cell surface RAGE–heparan sulphate interactions that lead to dissociation of RAGE homodimer (Figure 3A).

### 3.2. Sulphated K5 Polysaccharides Inhibit RAGE Binding to Amyloid β-Peptide and HMGB1

Heparin that has a high content of sulphated L-iduronic acid units binds to RAGE and inhibits RAGE–ligand binding and dimerization (Figure 2B and [1]). Epimerization of carbon C5 of D-glucuronic acid to create L-iduronic acid is not required for RAGE inhibition by sulphated oligosaccharides [43]. Capsular polysaccharide K5 derived from *E. coli* contains N-acetylglucosamine and glucuronic acid structures [44]. Here, we studied whether semi-synthetic non-epimerized sulphated K5 polysaccharides inhibit RAGE–ligand binding. O- and N-sulphated K5 oligosaccharides were able to inhibit RAGE binding to amyloid β-peptide and HMGB1 (Table 1) at similar concentrations that have previously been shown for heparin [1,45]. The minimum size of oligosaccharides required for strong inhibition was 3100–5800 Da. Sulphated disaccharides derived from chondroitin and dermatan sulphates did not inhibit RAGE–ligand binding (data not shown). These results are in line with the results from previous studies where the polymeric structure of sulphated carbohydrates was shown to be essential for inhibition of RAGE–ligand binding and RAGE dimerization [1,2]. Our results indicate that *E. coli* derived K5 polysaccharides can be used to design new RAGE targeting drug molecules (Figure 3A).

### 3.3. Glycyrrhizin and Heparin Inhibit AGE–RAGE -Interactions

Glycyrrhizin has two non-sulphated D-glucuronic acids in its structure and it inhibits RAGE binding to HMGB1 [46]. The inhibition is mainly due to binding of glycyrrhizin to HMGB1. However, glycyrrhizin shows minimal binding on RAGE. In addition, glycyrrhizin interferes HMGB1 binding to CXCR4, Toll-like receptor 2, and Toll-like receptor 4 [25,28,38]. Glycyrrhizin might be a potential molecule for RAGE targeting drug development because it can be easily modified [21,22]. Here, we tested whether glycyrrhizin affects homophilic interactions of RAGE in cell culture conditions using the PCA assay. Surprisingly, glycyrrhizin inhibited full-length luciferase activity in cells, that is, it was incompatible with the assay, thereby preventing further studies with glycyrrhizin in the PCA assay (data not shown).

**Figure 4:**
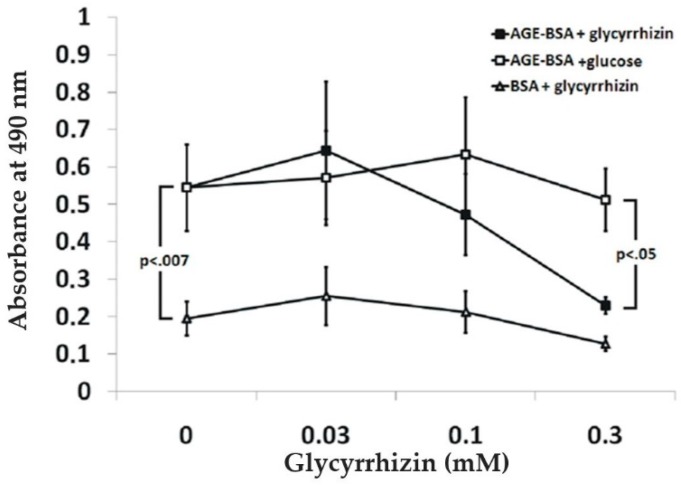
Glycyrrhizin inhibits RAGE binding to advanced glycation end product bovine serum albumin (AGE-BSA). Soluble RAGE binds specifically to AGE-BSA coated plastic wells. The binding was inhibited by glycyrrhizin. N ≥ 3; error bars represent ±SD.

**Figure 5:**
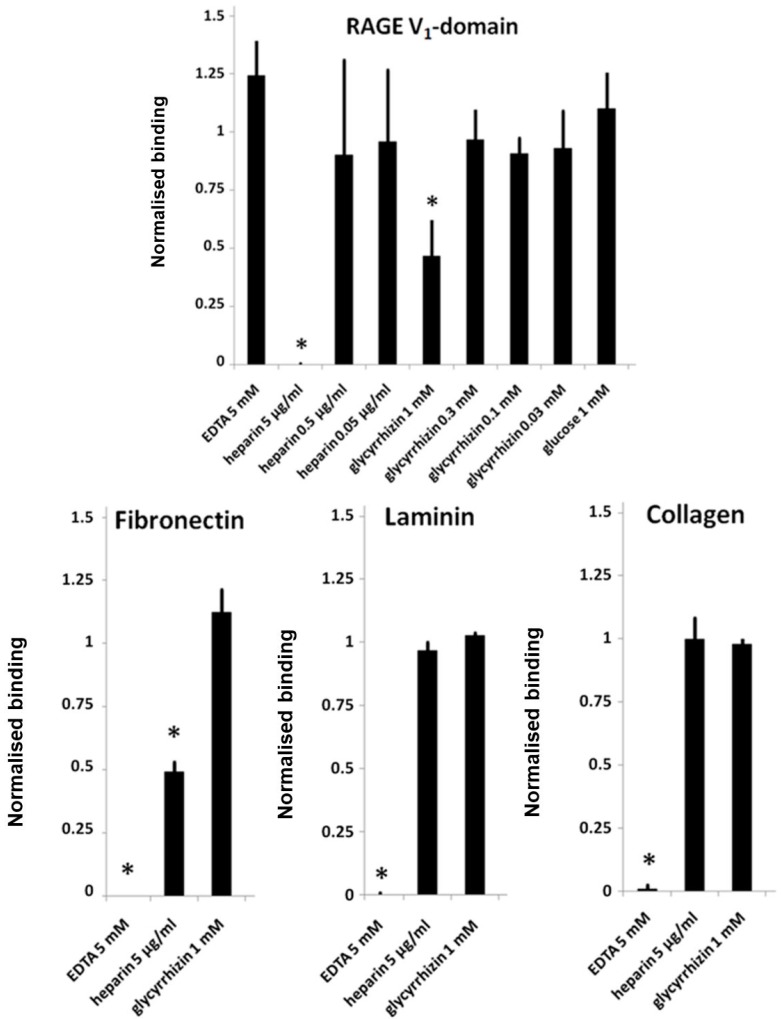
Glycyrrhizin and heparin inhibit cell surface AGE-mediated adhesion of PC3 prostate cancer cells to RAGE V_1_-domain. Microwells were coated with RAGE V_1_–Fc, type I collagen, laminin, or fibronectin (10 μg/mL) and blocked with BSA. Cells with possible inhibitors in serum free media were added to wells and adhered for 30 min at 37 °C, and unbound cells were washed away. Adherent cells were quantitated with an intracellular acidic phosphatase assay. Uninhibited adhesion was defined as 1. N = 3–5; Error bars represent ±SD; * = *p* < 0.05 when compared with uninhibited control.

AGE-BSA binding to RAGE induces receptor dimerization and signaling [47]. Here, we studied whether glycyrrhizin interferes with binding of RAGE to AGE-BSA. In a microwell binding assay, 300 µM glycyrrhizin inhibited RAGE binding to AGE-BSA coated microwells (Figure 4). The effect of glycyrrhizin on RAGE–AGE interactions was also tested in cell adhesion assay. PC3 cells express endogenously synthetized AGEs on their surface, which mediate cell binding to RAGE [40,48,49]. PC3 cells adhered to RAGE V_1_-domain, but not to C_1_- or C_2_-domains ([40]; data not shown). Glycyrrhizin at 1 mM concentration inhibited PC3 cell binding to RAGE V_1_-domain, but not to the control proteins fibronectin, laminin, or collagen. Furthermore, heparin inhibited cell binding to both RAGE V_1_-domain and fibronectin, but not to laminin and collagen. In contrast, EDTA inhibited cell binding to fibronectin, laminin, and collagen, but not to RAGE V_1_-domain (Figure 5). The finding that the cell binding to RAGE occurs independently of divalent cations excludes the role of Mac-1 integrin in the binding [45]. Our results indicate that AGE binding to RAGE can be inhibited by glycyrrhizin and heparin (Figure 3A).

### 3.4. Glycyrrhizin Inhibits HMGB1 Heparin Binding

HMGB1 signals to cells via mechanisms that are, at least partly, dependent on RAGE–heparan sulphate interactions [1,27,28,50]. Whether binding of HMGB1 to sulphated glycosaminoglycans is necessary to RAGE–HMGB1 signaling is currently poorly understood [2,51]. Described heparin-binding sites of HMGB1 are located at the linker sequence between A- and B-boxes and at the amino terminal part of the A-box [2,52,53]. Interestingly, soluble HMGB1 A-box, which lacks RAGE binding site but has a heparin-binding site, inhibits cell migration [54,55,56,57]. This suggests that HMGB1 binding to HSPGs might be necessary to RAGE–HMGB1 complex signaling. Here, we studied the effect of glycyrrhizin on HMGB1 heparin binding. First, we tested whether A-box binds to heparin in a similar manner to the full-length HMGB1. The A-box bound to a heparin–Sepharose in column chromatography and was eluted with the same NaCl concentration (~0.7 M) as full-length HMGB1 (data not shown). This suggests that the A-box lacking heparin binding cationic amino acid residues in a linker sequence is still able to bind to heparin.

**Figure 6:**
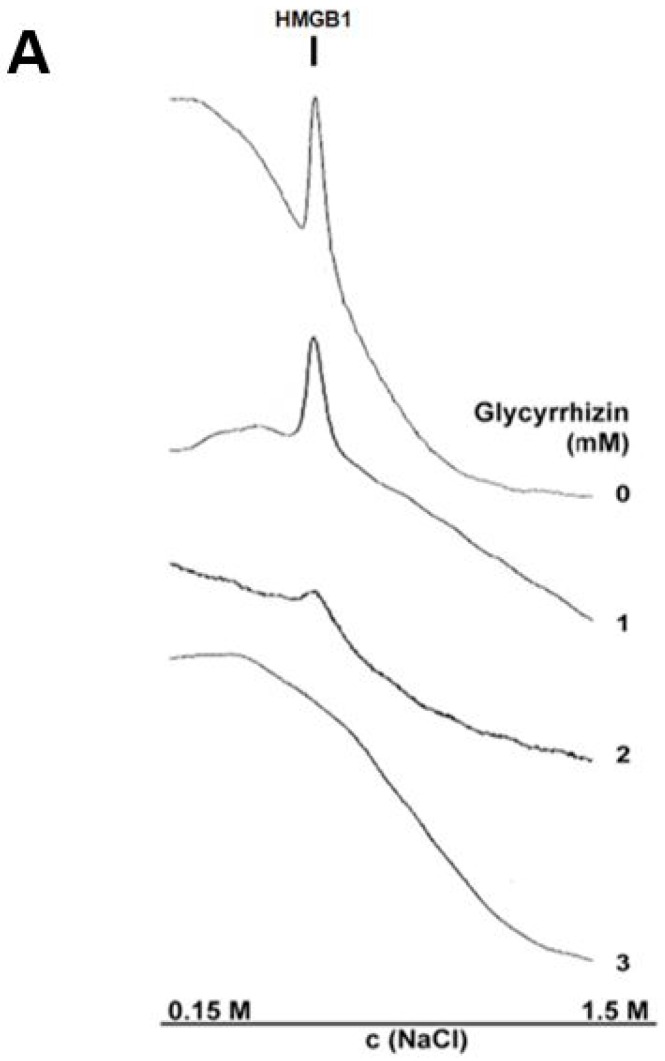

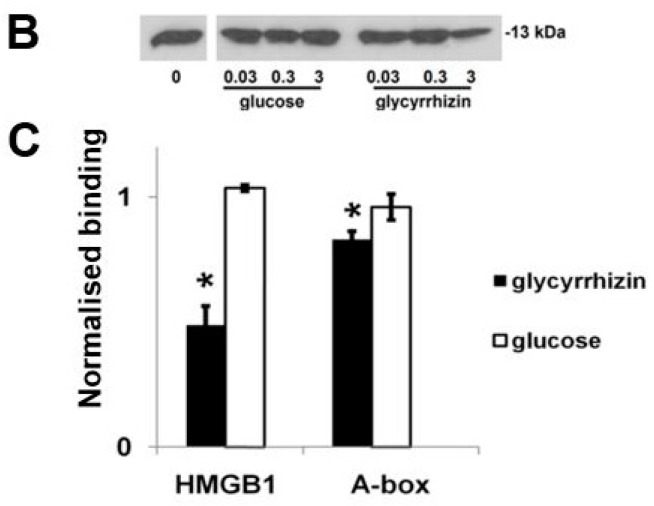
Glycyrrhizin inhibits HMGB1 binding to heparin. (**A**) HMGB1 in phosphate buffered saline (PBS) was loaded to a heparin–Sepharose column in the presence of 0–3 mM glycyrrhizin, and bound protein was eluted with increased concentrations of NaCl. The eluted protein was monitored by measuring the absorbance at 280 nm. Chromatograms indicate that HMGB1 binding to heparin–Sepharose was inhibited by increasing concentrations of glycyrrhizin and was totally abolished with 3 mM glycyrrhizin. (**B**) HMGB1 A-box binds to heparin and binding is inhibited by glycyrrhizin. A-box was incubated with heparin–Sepharose, and bound protein was precipitated and analyzed in Western blotting. A-box of HMGB1 bound to heparin–Sepharose and the binding was specifically inhibited with 3 mM glycyrrhizin. Glucose served as a negative control. N = 3; Error bars represent ±SD. (**C**) Glycyrrhizin inhibits binding of full-length HMGB1 and HMGB1 A-box to heparin–Sepharose. HMGB1 or A-box in PBS was precipitated with heparin–Sepharose in the presence of 3 mM glycyrrhizin or glucose. Precipitated HMGB1 was detected with Coomassie blue stained SDS-PAGE and precipitated A-box was detected with anti-HMGB1 Western blotting. Optical densities of protein bands were measured and optical densities of uninhibited samples were determined as 1. N = 3, error bars represent ±SD; * = *p* < 0.05 when compared with glucose control.

Next, we tested whether glycyrrhizin can inhibit heparin binding in heparin–Sepharose chromatography. The excess of glycyrrhizin prevented HMGB1 binding to the chromatography column (Figure 6A). Further, a heparin–Sepharose column bound HMGB1 was eluted with a glycyrrhizin gradient at the same concentration of glycyrrhizin as the control protein thrombin ([58]; data not shown). Next, we studied the effect of glycyrrhizin on heparin binding of full-length HMGB1 and A-box using a heparin–Sepharose precipitation assay. Glycyrrhizin inhibited binding of both the full-length HMGB1 and the A-box (Figure 6B,C). These results indicate that the A-box and the full length HMGB1 bind to heparin in a similar manner, and that their binding to heparin is inhibited by millimolar concentrations of glycyrrhizin. It is tempting to speculate that glycyrrhizin might inhibit RAGE signaling via interfering HMGB1–heparan sulphate interactions (Figure 3B).

Our results show that RAGE dimerization occurring in living cells can be inhibited by heparin, RAGE ligand binding can be inhibited by sulphated K5 polysaccharides, and RAGE–AGE interactions and HMGB1 binding to heparin can be inhibited by glycyrrhizin. We conclude that sulphation of D-glucuronic acid and L-iduronic acid structures offer a promising strategy for the development of RAGE targeting molecules.

## Figures and Tables

**Table 1:** Sulphated K5 polysaccharides inhibit receptor for advanced glycation end products (RAGE)–ligand binding. Binding of soluble biotinylated ligands to full length extracellular human RAGE (sRAGE) coated plastic microwells was analysed as described in materials and methods. Representative IC50 values are shown. NS = N-sulphated, OS = O-sulphated, OS(H) = highly O-sulphated, 3100 = size of 3100 Da, 5800 = size of 5800 Da. ND = not determined.

Polusaccharide	Amyloid β-Peptide	HMGB1
**K5**	6837 µg/mL	ND
**K5 NS**	34.7 µg/mL	ND
**K5 OS (H)**	0.9 µg/mL	ND
**K5 NS, OS (H)**	1.5 µg/mL	1.0 µg/mL
**K5 NS, OS (H) 3100**	21.3 µg/mL	0.9 µg/mL
**K5 NS, OS (H) 5800**	1.6 µg/mL	0.9 µg/mL
